# Vomiting of Fluids but Not of Solids

**Published:** 1896-01

**Authors:** W. A. Yingling

**Affiliations:** Ness City, Kansas


### VOMITING OF FLUIDS BUT NOT OF SOLIDS.

NESS CITY, KANSAS, January 10th, 1896.

EDITOR OF THE HOMŒOPATHIC PHYSICIAN :—Apropos of that symptom of Bismuth, vomiting of liquids but not of solids, referred to in the December number at page 563, I find in my private repertory: "Child vomits all fluids at once, BISM., *Zinc.*;" "Vomits water and all fluids when reaching. Can eat his food for several days, then vomits and keeps it up for a whole day, *Bism.*;" "Vomiting immediately of water drunk, but not food, BISM., *Zinc*, etc." Dr. Ad. Lippe recognized this as a key-note of Bismuth, as you will find in THE HOMŒOPATHIC PHYSICIAN, Vol. XIV, p. 101, where he says, "*the curative effects have been confirmed—only water thrown up while other substances entering the stomach are retained.*" Eup-perf., Nux, Alum., etc., have very similar symptoms.

Very truly yours,

W. A. YINGLING.

